# The Micro-RNA Cargo of Extracellular Vesicles Released by Human Adipose Tissue-Derived Mesenchymal Stem Cells Is Modified by Obesity

**DOI:** 10.3389/fcell.2021.660851

**Published:** 2021-05-20

**Authors:** Alfonso Eirin, Yu Meng, Xiang-Yang Zhu, Yongxin Li, Ishran M. Saadiq, Kyra L. Jordan, Hui Tang, Amir Lerman, Andre J. van Wijnen, Lilach O. Lerman

**Affiliations:** ^1^Department of Medicine, Division of Nephrology and Hypertension, Mayo Clinic, Rochester, MN, United States; ^2^Department of Nephrology, The First Hospital Affiliated to Jinan University, Guangzhou, China; ^3^Department of Cardiovascular Medicine, Mayo Clinic, Rochester, MN, United States; ^4^Department of Orthopedic Surgery, Mayo Clinic, Rochester, MN, United States

**Keywords:** exosomes, mesenchymal stem cells, microvesicles, miRNA, obesity

## Abstract

Obesity is a chronic disease that interferes with normal repair processes, including adipose mesenchymal stem/stromal cells (ASCs) function. ASCs produce extracellular vesicles (EVs) that activate a repair program in recipient cells partly via their micro-RNA (miRNA) cargo. We hypothesized that obesity alters the miRNA expression profile of human ASC-derived EVs, limiting their capacity to repair injured cells. Human ASCs were harvested from obese and age- and gender-matched non-obese (lean) subjects during bariatric or cosmetic surgeries, respectively (*n* = 5 each), and their EVs isolated. Following high-throughput sequencing analysis, differentially expressed miRNAs were identified and their gene targets classified based on cellular component, molecular function, and biological process. The capacity of human lean- and obese-EVs to modulate inflammation, apoptosis, as well as mitogen-activated protein kinase (MAPK) and Wnt signaling in injured human proximal tubular epithelial (HK2) cells was evaluated *in vitro*. The number of EVs released from lean- and obese-ASCs was similar, but obese-EVs were smaller compared to lean-EVs. Differential expression analysis revealed 8 miRNAs upregulated (fold change > 1.4, *p* < 0.05) and 75 downregulated (fold change < 0.7, *p* < 0.05) in obese-EVs vs. lean-EVs. miRNAs upregulated in obese-EVs participate in regulation of NFk-B and MAPK signaling, cytoskeleton organization, and apoptosis, whereas those downregulated in obese-EVs are implicated in cell cycle, angiogenesis, and Wnt and MAPK signaling. Treatment of injured HK2 cells with obese-EVs failed to decrease inflammation, and they decreased apoptosis and MAPK signaling significantly less effectively than their lean counterparts. Obesity alters the size and miRNA cargo of human ASC-derived EVs, as well as their ability to modulate important injury pathways in recipient cells. These observations may guide development of novel strategies to improve healing and repair in obese individuals.

## Introduction

Adipose mesenchymal stem/stromal cells (ASCs), multipotent cells with capacity for self-renewal and differentiation, emerged as a promising clinical cell-based therapy because of their potential for autologous transplantation. These cells possess important pro-angiogenic and immunomodulatory properties, can be obtained in large amounts from several tissues, including adipose tissue, and their delivery has shown potential efficacy for the treatment of several diseases (Dominici et al., 2006).

ASCs release multiple extracellular vesicles (EVs), which play an important role in mediating ASC paracrine function (Lotvall et al., 2014). ASC-derived EVs carry genetic and protein content capable of promoting repair in recipient cells (Lai et al., 2011; Yeo et al., 2013). Furthermore, many protective effects of ASC-derived EVs have been attributed to their cargo of micro-RNAs (miRNAs), non-coding RNA fragments that function as post-transcriptional regulators of gene expression. In line with this, we have previously shown that EVs isolated from porcine adipose tissue-derived ASCs contain multiple miRNAs capable of modulating several cellular pathways, including angiogenesis, cellular transport, apoptosis, and proteolysis (Eirin et al., 2014).

However, experimental data suggest that cardiovascular risk factors may alter the miRNA content of ASC-derived EVs, limiting their capacity to repair damaged tissues. We have shown that EVs isolated from adipose tissue ASCs of obese pigs contain miRNAs that modulate pathways involved in the development of obesity, diabetes, and insulin signaling (Meng et al., 2018). Furthermore, we found that obesity alters the cargo of mitochondria-related miRNAs (Farahani et al., 2020) as well as miRNAs capable of targeting several pro-angiogenic genes in swine ASC-derived EVs (Eirin et al., 2020). Importantly, intra-renal delivery of porcine obese-EVs failed to preserve the microvasculature and improve function in pigs with renovascular disease (Eirin et al., 2020; Song et al., 2020), suggesting that swine obesity-induced changes in the miRNA content of EVs might exert important post-transcriptional changes in recipient cells, and in turn impair the reparative capacity of EVs. Yet, whether obesity modulates the miRNA content of human ASC-derived EVs remains to be clarified. Thus, the current study tested the hypotheisis that obesity alters the miRNA cargo of ASC-derived EVs, and limits their capacity to repair injured renal tubular cells.

## Materials and Methods

Studies were performed in EVs isolated from adipose tissue-derived ASCs harvested from obese and age- and gender-matched non-obese (lean) subjects during bariatric or cosmetic surgeries, respectively at the First Hospital Affiliated to Jinan University, Guangdong, China (IRB number: [2018] 048). Informed written consent was obtained after receiving approval from the Institutional Research Ethics Committee. Our studies adhered to the standard biosecurity of the First Hospital Affiliated to Jinan University. Entry criteria for obese patients included age > 18 years old and body mass index (BMI) > 30 kg/m^2^, whereas exclusion criteria included heavy smoking, drug abuse, cancer, severe heart valve or systemic inflammatory diseases (asthma, chronic peptic ulcer, tuberculosis, rheumatoid arthritis, periodontitis, ulcerative colitis, Crohn’s disease, sinusitis, active hepatitis, type-I Diabetes, thyroid autoimmune disease, or any acute inflammatory state associated with bacterial or viral infection). Entry criteria for lean controls included age > 18 years, BMI < 25 kg/m^2^, and healthy overall state, whereas exclusion criteria included heavy smoking and drug abuse. Blood samples were collected in all patients and fasting blood sugar, fasting insulin, hemoglobin A1C (HbA1c), total cholesterol, uric acid, serum creatinine, cystatin-C, plasma renin activity (PRA), and C-reactive protein (CRP) were assessed by standard procedures.

### ASCs and EV Harvesting

ASCs were isolated from 5–10 g of subcutaneous abdominal fat, digested in collagenase-H, filtered with 0.2 mm syringe filter, and cultured for 3 about weeks in advanced minimal essential medium (GIBCO/Invitrogen, Grand Island, NY, United States) with 5% platelet lysate (PLTmax^®^, Mill Creek Life Sciences, Rochester, MN) in 37°/5% CO_2_, as previously described (Crespo-Diaz et al., 2011). Cells were characterized using flow cytometry (Amnis FlowSight high-speed cellular imaging, Millipore) for the expression of ASC markers (CD73 +, CD90 +, CD105 +, CD14−, CD34−, and CD45−), and trans-differentiation into chondrocytes, adipocytes, and osteocytes, as previously described (Ebrahimi et al., 2013). The third passage was collected and EVs isolated from supernatants of ASCs by ultracentrifugation. Briefly, the conditioned medium of 107 ASCs was centrifuged to remove debris. Cell-free supernatants were then subjected to a second ultra-centrifugation, washed with M199, and centrifuged one more time (see Supplementary Material). Following the standards described by Minimal information for studies of extracellular vesicles 2018 (MISEV2018) guidelines (Thery et al., 2018), EVs were characterized based on the expression of common EV (CD9, CD63, and CD81) protein markers (western blotting), transmission electron microscopy (TEM negative staining, JEOL 1200 EXII), and nanoparticle tracking analysis (NTA, NanoSight NS300) to assess EV concentration and size distribution (Filipe et al., 2010).

### miRNA Sequencing

The EV miRNA cargo in all samples was assessed by miRNA sequencing, as described (Li et al., 2020; see Supplementary Material), after passing quality control tests (Supplementary Tables 1, 2). Total RNA was extracted from EVs using the exoRNeasy Maxi Kit (cat#77044, Qiagen, Germany). Libraries for small RNA sequencing were constructed before miRNA sequencing following the QIAseq miRNA Library Kit (cat#331505, Qiagen, Germany) standard protocol. The library was constructed in the GenCoding Lab (Guangzhou, China), and the sequencing was performed in the Haplox Biotechnology Lab (Shenzhen, China) using an Illumina NGS system (MiSeq Personal Sequencer, NextSequence500, HiSeq 2500). Data were analyzed with CLC (Biomedical) Genomics Workbench. Unaligned FASTQs were used to generate aligned BAMs, raw and normalized known mature miRNA expression counts and predicted novel miRNAs, which were expressed as normalized total reads. Differential expression analysis was performed with edgeR2.6.2. miRNAs with fold-change (obese-EVs/lean-EVs) > 1.4 (log2 = 0.5) were considered upregulated, whereas those with fold-change < 0.7 (log2 = −0.5) were considered downregulated in obese-EVs vs. lean-EVs. Differential *p*-values were FDR-corrected using the Benjamini-Hochberg-Yekutieli procedure (Kim and van de Wiel, 2008).

TargetScan7.2^1^ and MiRWalk 2.0 (Sticht et al., 2018) were used to identify genes targeted by miRNAs dysregulated in obese-EVs. Functional enrichment analysis of miRNA target genes was performed using Gene Set Enrichment Analysis (GSEA) (Subramanian et al., 2005), and genes were classified by molecular function, cellular component, and biological process.

### miRNA Validation

To validate the miRNA profile enclosed only within human EVs to remove any external adhering RNA, we treated lean- and obese-EVs with 0.2 μg/ml of RNAse A (Thermo Fisher Scientific) for 30 min at 37°C, and measured expression of randomly selected candidates by quantitative-polymerase chain reaction (qPCR).

### EV Functional Studies

To explore the functional implications of obesity-induced changes in the EV cargo, we compared the capacity of human lean- and obese-EVs in human proximal tubular epithelial cells (HK2 cells) to modulate inflammation, apoptosis, as well as mitogen-activated protein kinase (MAPK) and Wnt signaling, important processes identified in miRNA sequencing. We used HK2 cells because obesity frequently coexists with and is associated with poor outcomes in kidney disease (Martins et al., 2010). HK2 were co-incubated with 10 ng/ml tumor necrosis factor (TNF)-α and 10 μM antimycin-A (AMA) for 24 h, a model that mimics renal ischemic injury *in vitro* (Zhang et al., 2013; Liang et al., 2014), and injured HK2 cells were then co-cultured for 24 h with a pool of 10 μg/ml of either lean- or obese-EVs harvested from different subjects (∼1 × 10^10^ EVs/ml). All experiments were performed in triplicate. Inflammation was assessed by nuclear translocation of the pro-inflammatory transcription factor nuclear factor (NF)-kB, as previously described (Pawar et al., 2019). Immunofluorescent staining of HK2 cells for NF-kB (abcam, 1:200, Cambridge, MA, United States) and 4’,6’-diamino-2-phenylindole (DAPI, Thermo Fisher Scientific, Waltham, MA) was performed. Nuclear and cytoplasmic localization was assessed and double positive (NFkB^+^/DAPI^+^) areas quantified using a computer-aided image analysis program (ZEN^®^ 2012 blue edition; Carl Zeiss SMT, Oberkochen, Germany). HK2 cell apoptosis was evaluated by terminal deoxynucleotidyl transferase dUTP nick end labeling (TUNEL) staining (abcam, cat#, ab21171, Cambridge, MA) (Eirin et al., 2018). Apoptotic cells were identified under fluorescence microscopy by co-staining with nuclear (DAPI, blue) and TUNEL (green), manually counted (20 images/sample), and results from all fields averaged. Expression of phosphorylated p38 (p-p38) MAPK and WNT-1 was assessed by Western blotting (Cell signaling, cat#: 9212, 1:1,000 and abcam cat#: ab15251, 1:100, respectively).

### Statistical Analysis

Statistical analysis was performed using JMP software package version 14 (SAS Institute, Inc., Cary, NC, United States). Results were expressed as mean + SD. The Shapiro–Wilk test was used to test for deviation from normality. Parametric (Student’s *t*-test) and non-parametric (Kruskal-Wallis) tests were used as appropriate, and significance was accepted for *p* < 0.05.

## Results

### Systemic Characteristics

Table 1 shows the clinical, laboratory, and demographic characteristics of lean and obese subjects included in the study (*n* = 5 each). Gender and age did not differ between the groups, whereas BMI and waistline circumference were higher in obese compared to lean individuals, as was mean blood pressure. Fasting blood sugar levels and HbA1c were similar in lean and obese subjects, but fasting insulin levels were higher in obese individuals. Total cholesterol, serum creatinine, and CRP levels were comparable between lean and obese participants, whereas uric acid, cystatin-c, and PRA levels were elevated in obese patients, likely reflecting obesity-induced hyperfiltration and renal damage, respectively.

**TABLE 1:** Systemic characteristics in Lean and Obese subjects (*n* = 5, each).

**Parameter**	**Lean**	**Obese**
Gender (female/male)	3/2	3/2
Age (years)	24.5 ± 2.8	26.2 ± 6.1
Body Mass Index (kg/m^2^)	19.4 (18.4–20.1)	50.1 (40.6–72.6)*
Waistline circumference (cm)	61.0 (59.5–67.0)	139.2 (129.3–171.0)*
Mean blood pressure (mmHg)	78.8 ± 6.1	113.9 ± 8.*
Fasting blood sugar (mg/dl)	5.0 (4.3–5.3)	7.1 (4.6–8.9)
Fasting insulin (mIU/L)	13.3 (11.9–17.3)	42.9 (21.5–64.7)*
HbA1c (mmol/mol)	5.2 (5.0–5.5)	6.5 (5.2–7.9)
Total cholesterol (mmol/L)	4.4 ± 0.3	5.2 ± 0.9
Uric acid (μmol/L)	289.5 (271.3–328.1)	546.2 (429.3–668.9)*
Serum creatinine (μmol/L)	55.8 ± 16.7	58.8 ± 22.4
Cystatin-C (mg/L)	0.9 (0.8–0.9)	1.2 (0.9–1.5)*
PRA (ng/ml/h)	0.6 (0.4–0.7)	3.2 (1.9–3.7)*
CRP (mg/L)	3.7 (2.5–4.7)	7.3 (2.5–12.1)

*p ≤ 0.05 vs. Lean. HB1AC, hemoglobin A1C; PRA, plasma renin activity; CRP, C-reactive protein. Normal distributed data were expressed as mean + SD and compared used Parametric (Student’s t-test) tests, whereas data that did not followed a normal distribution were expressed as median (range) and compared using non-parametric (Kruskal-Wallis) tests. Significance for all tests was accepted for p < 0.05.

#### ASC and EV Characterization

**FIGURE 1:**
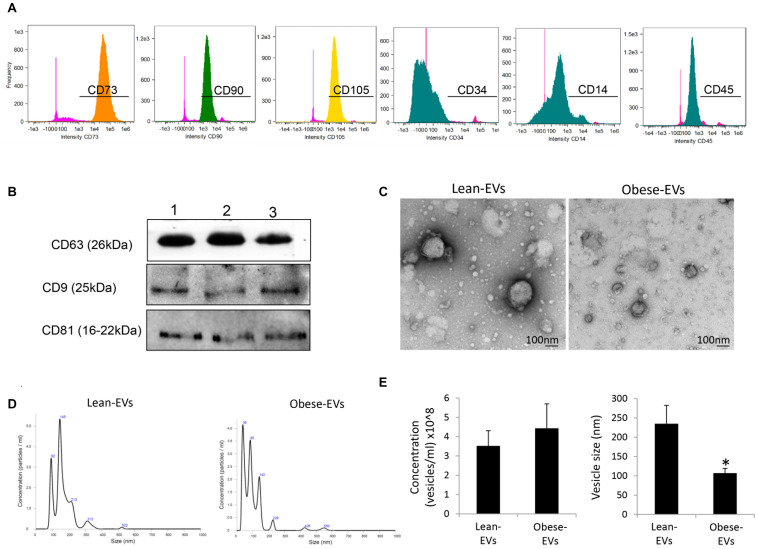
Characterization of ASC-derived EVs and their daughter EVs. **(A)** Flow cytometry analysis of human ASCs. Cells were stained with anti-CD73, CD90, CD105, CD14, CD34, and CD45 antibodies. **(B)** ASC-derived EVs expressed common EV markers, including CD9, CD63, and CD81 by Western blotting. **(C)** Transmission electron microscopy showing ASC-derived lean- and obese-EVs exhibiting the classic “cup-like” morphology. **(D)** Representative size-distribution curve of lean- and obese-EVs by nanosight tracking analysis (NTA). **(E)** Quantification of EV concentration and size by NTA (Student’s *t*-test). *p < 0.05 vs. Lean-EVs.

Flow cytometry analysis confirmed that ASCs expressed common ASC markers including CD73, CD90, and CD105, but did not express CD14, CD34, and CD45 (Figure 1A). ASC-derived EVs expressed common EV markers, including CD9, CD63, and CD81 (Figure 1B) and exhibited the classic “cup-like” morphology on transmission electron microscopy (Figure 1C). The number of EVs released from lean- and obese-ASCs was similar, but obese-EVs were smaller compared to lean-EVs (Figures 1D,E).

#### Identification and Functional Analysis of Differentially Expressed miRNAs

**FIGURE 2:**
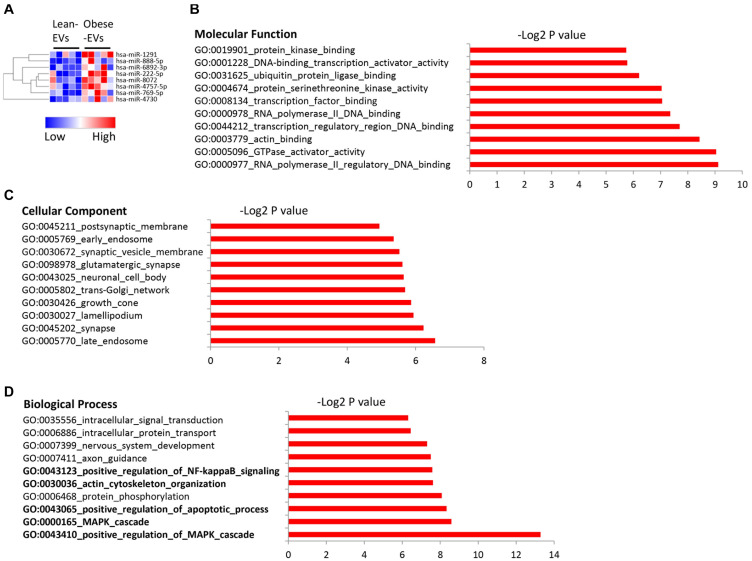
Upregulated miRNA in human ASC-derived EVs and their gene targets. Heat map of miRNAs upregulated in obese-EVs vs. lean-EVs **(A)**. GSEA analysis of the molecular function **(B)**, cellular component **(C)**, and biological process **(D)** of gene targets of miRNAs upregulated in obese-EVs.

A total of 2,464 miRNAs were mapped, among which 8 were upregulated in obese-EVs vs. lean-EVs (Figure 2A). Supplementary Table 3 shows the entire list of target genes of miRNAs upregulated in obese-EVs. Molecular function analysis of these target genes revealed avid transcription factor and GTPase activator activity, as well as ubiquitin and actin binding activity (Figure 2B). These miRNAs are capable of targeting genes equally distributed in cellular and membrane compartments, including endosome, synapse, Golgi apparatus, and synaptic vesicle membrane (Figure 2C). Functional clustering analysis of these miRNA targets indicated that the most prominent gene ontology categories include regulation of MAPK and apoptotic process, actin and cytoskeleton organization, and regulation of NF-kB signaling (Figure 2D).

**FIGURE 3:**
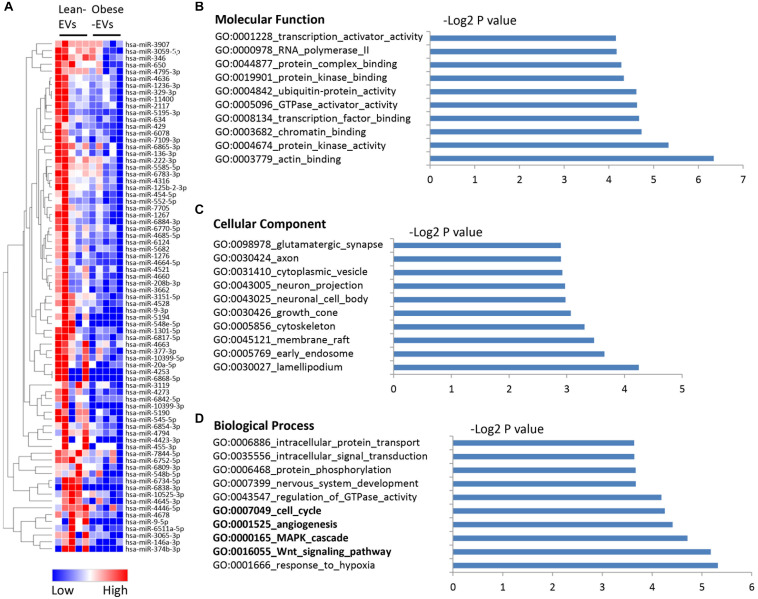
Downregulated miRNA in human ASC-derived EVs and their gene targets. Heat map of miRNAs downregulated in obese-EVs vs. lean-EVs **(A)**. GSEA analysis of the molecular function **(B)**, cellular component **(C)**, and biological process **(D)** of gene targets of miRNAs upregulated in obese-EVs.

We also identified 75 miRNAs downregulated in obese-EVs compared to lean-EVs (Figure 3A). Supplementary Table 4 shows the entire list of target genes of miRNAs downregulated in obese-EVs. Target genes of these miRNAs have protein kinase, actin, GTPase activator, and chromatin binding activity (Figure 3B), and are again similarly distributed between cellular and membrane compartments, such as endosome, cytoskeleton, and cytoplasmic vesicles (Figure 3C). These target genes are primarily involved in modulation of cell cycle, angiogenesis, as well as Wnt and MAPK signaling (Figure 3D).

**FIGURE 4:**
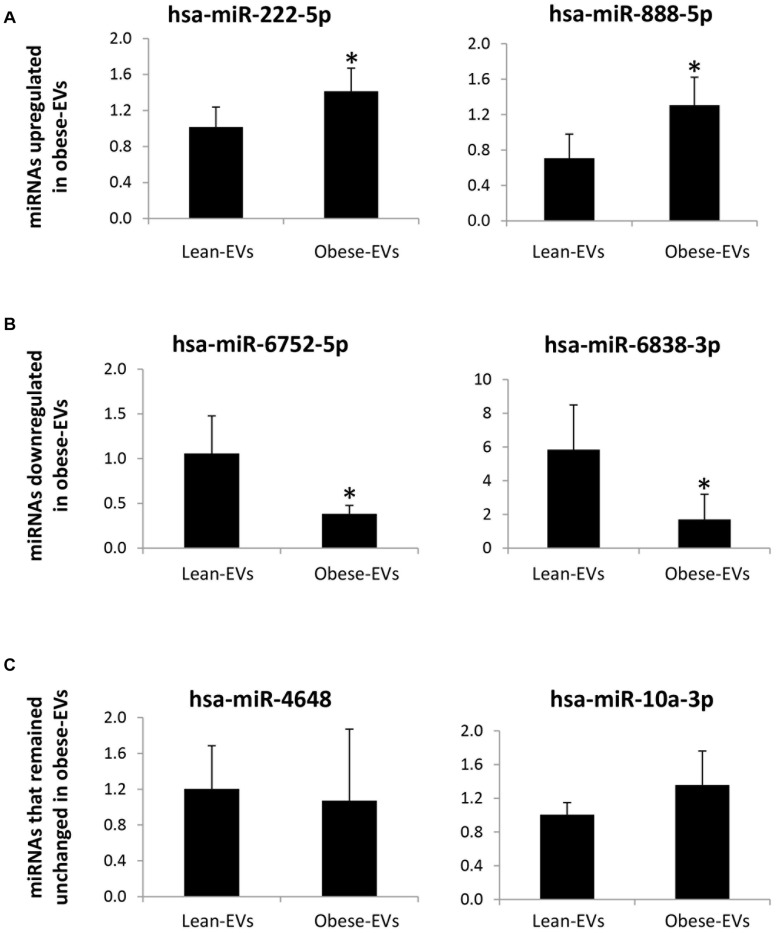
miRNA sequencing validation. In samples treated with RNAse expression of candidate miRNAs by qPCR followed the same patterns as the original miRNA sequencing findings (without RNAse treatment). hsa-miR-222-5p and has-miR-888-5p, which were upregulated in obese-EVs vs. lean-EVs by miRNA sequencing analysis were also upregulated in obese-EVs vs. lean-EVs treated with RNAse **(A)**. hsa-miR-6752-5p and has-miR-6338-3p, which were downregulated in obese-EVs vs. lean-EVs by miRNA sequencing analysis were also downregulated in obese-EVs vs. lean-EVs treated with RNAse **(B)**. hsa-miR-4648 and has-miR-10a3p, which did not differ between obese-EVs and lean-EVs by miRNA sequencing analysis remained similar in obese-EVs and lean-EVs treated with RNAse **(C)** (all Student’s *t*-test). *p < 0.05 vs. Lean-EVs.

In a subset of samples treated with RNAse, expression of candidate miRNAs followed the same patterns as the original miRNA sequencing findings (in RNAse-untreated EVs). Specifically, hsa-miR-222-5p and has-miR888-5p were upregulated (Figure 4A), and hsa-miR-6752-5p and has-miR6838-3p were downregulated (Figure 4B) in obese-EVs vs. lean-EVs, whereas expression of hsa-miR-4648 and has-miR-10a-3p did not differ between the groups (Figure 4C).

#### EV Functional Studies

**FIGURE 5:**
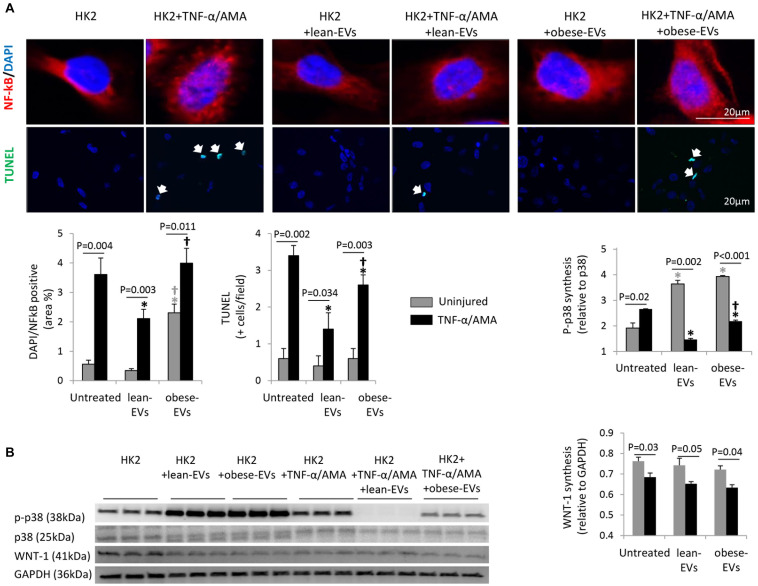
Effects of lean- and obese-EVs on pathways detected in RNA sequencing. Representative immunofluorescence staining of HK2 cells with NFk-B/DAPI and TUNEL, and quantification of NFk-B/DAPI co-localization and the number of TUNEL + cells **(A)** (Student’s *t*-test). HK2 cells were co-incubated with 10 ng/ml TNF-α and 10μM AMA for 24 h and subsequently treated with human lean- or obese-EVs. Western blot quantification of p-p38 and WNT-1 synthesis in study groups **(B)** (Student’s *t*-test). ^∗^*p* < 0.05 vs. HK2, ^†^*p* < 0.05 vs. HK2 + lean-EVs.

Treatment of HK2 cells with TNF-α and AMA increased nuclear translocation of NF-kB (NF-kB/DAPI co-localization), apoptosis (TUNEL + cells) (Figure 5A), and MAPK signaling (p-p38 expression) (Figure 5B), but decreased Wnt signaling (WNT-1 expression).

Treatment of uninjured HK2 cells with lean-EVs did not affect nuclear NF-kB translocation, apoptosis, or Wnt signaling, but increased p-p38 expression. Contrarily, treatment of uninjured HK2 cells with obese-EVs increased nuclear translocation of NF-kB compared to uninjured untreated HK2 cells and uninjured HK2 cells treated with lean-EVs, did not affect apoptosis or Wnt signaling, and increased p-p38 expression compared to uninjured HK2 cells.

Treatment of injured HK2 cells with lean-EVs decreased nuclear NF-kB translocation, apoptosis (TUNEL + cells), and MAPK signaling (p-p38 expression) compared to injured untreated HK2 cells. However, treatment of injured HK2 cells with obese-EVs failed to decrease nuclear translocation of NF-kB. Although obese-EVs decreased the number of TUNEL + cells and p-p38 expression, the decrease was significantly less effective than their lean counterparts. WNT-1 expression in injured HK2 cells remained unaltered after treatment with either lean- or obese-EVs.

## Discussion

The current study shows that obesity alters the size and miRNA cargo of human ASC-derived EVs. The number of EVs released from lean- and obese-ASCs was similar, but obese-EVs were smaller compared to lean-EVs, in line with our previous observations in swine obese-EVs (Conley et al., 2019). miRNAs upregulated in obese-EVs target preferentially clusters of genes participating in regulation of NF-kB and MAPK signaling, cytoskeleton organization, and apoptosis, whereas miRNAs downregulated in obese-EVs target groups of genes implicated in cell cycle, angiogenesis, and MAPK and Wnt signaling. Furthermore, treatment of injured human renal tubular cells with obese-EVs failed to decrease inflammation and exerted lower anti-apoptotic efficacy and MAPK signaling modulation compared to lean-EVs. These observations indicate that obesity alters packaging of miRNAs into human ASC-derived EVs, favoring inclusion of miRNAs involved in pro-inflammatory signaling and programed cell death and exclusion of those implicated in cell proliferation and angiogenic pathways. These alterations may in turn be linked to impaired ability of EVs to repair renal tubular cells *in vitro*.

Accumulating evidence suggests that EVs are pivotal mediators of the paracrine function of ASCs (Lai et al., 2011; Yeo et al., 2013). These membrane microparticles contain multiple genes and proteins, as well as miRNAs, which modulate gene expression in recipient cells by degradation of translational repression. We have previously shown that miRNAs enriched in porcine ASC-derived EVs are predicted to mediate post-transcriptional control of genes implicated in several cellular pathways, including angiogenesis, cellular transport, apoptosis, and proteolysis (Eirin et al., 2014). We have also shown that intra-renal infusion of swine ASC-derived EVs confers important protective effects in the post-stenotic pig kidney (Eirin et al., 2017, 2018). Furthermore, a previous clinical trial suggests that ASC-derived EV therapy is safe and effective in ameliorating renal inflammation and improving estimated glomerular filtration rate in patients with chronic kidney disease (Nassar et al., 2016), underscoring the potential of EVs to preserve renal function.

However, many potential candidates for autologous ASC-derived EV therapy are exposed to the deleterious effects of obesity, which interferes with many aspects of ASC biology and function (Meng et al., 2018). In this study, we took advantage of high-throughput sequencing analysis to characterize and compare the miRNA profile of EVs harvested from human lean- and obese- adipose tissue-derived ASCs, and further explored whether obesity-induced changes in the miRNA cargo interfere with their ability to repair injured tubular cells. We identified 8 miRNAs upregulated and 75 downregulated in obese-EVs vs. lean-EVs. Functional annotation clustering analysis of targets of miRNAs upregulated in obese-EVs indicated that these miRNAs are capable of modulating several cellular functions. Top functional categories of miRNA target genes include regulation of NF-kB signaling, a pro-inflammatory pathway that regulates immune response by activation of cytokines, chemokines, and adhesion molecules (Lawrence, 2009). In line with this, we observed that treatment with obese-EVs increased NF-kB nuclear translocation in both untreated and injured human renal tubular cells, suggesting that obesity may trigger a pro-inflammatory response in EV-recipient cells.

Upregulated miRNAs in obese-EVs can also target cytoskeleton genes, including genes involved in the control of cell shape and adhesion such as Palladin Cytoskeletal Associated Protein (*PALLD*), and genes involved in adherens junctions like Actin Binding LIM Protein Family Member 3 (*ABLIM3*). The cytoskeleton not only plays an important role in cellular repair (Abreu-Blanco et al., 2012), but also serves as a key effector and mediator of cell signaling (Moujaber and Stochaj, 2020). For example, cytoskeletal proteins can regulate the onset of programmed cell death pathways, such as apoptosis (Gourlay and Ayscough, 2005). In line with this notion, we found that miRNAs upregulated in obese-EVs are capable of targeting several genes involved in apoptosis, such as the BCL-2 Associated X-Apoptosis Regulator (*BAX*) and members of the caspase pathway, like caspase-2 (*CASP*2) and caspase-9 (*CASP9*). Interestingly, unlike the anti-apoptotic effect of lean-EVs, we found that the reduction in the number of TUNEL + cells was significantly less effectively achieved by obese-EVs than their lean counterparts, suggesting that obesity-induced changes in the miRNA cargo of EVs may be associated with limited ability to modulate apoptosis in target cells.

In contrast, top functional categories of target genes of miRNAs downregulated in obese-EVs include cell cycle and pro-angiogenic pathways. Cell cycle miRNA targets include genes involved in DNA repair (e.g., *BRCC3*, *LIG3*, and *DCLRE1A*), chromosome segregation (e.g., *MIS18A*, *RCC2*, and *MKI67*), and cell adhesion (e.g., *CSNK2A1*, *PARD3*, and *SRC*). Activation of cell cycle is a critical step in stem cell-induced tissue regeneration (Fan et al., 2020). In order to recover, injured cells must enter and progress through the cell cycle, which allows transcription of genes necessary for DNA replication and mitosis. In agreement with this concept, we have shown that EVs isolated from porcine lean-ASCs are enriched with several transcription factors involved in chromosome organization (Eirin et al., 2014) and increase proliferation of human umbilical endothelial cells (Eirin et al., 2020). However, increased cellular senescence, a state of permanent cell cycle arrest, is associated with impaired cellular proliferation (van Deursen, 2014).

miRNAs with lower expression in obese-EVs compared to lean-EVs can also target genes implicated in pro-angiogenic pathways. This includes genes participating in angiopoietin signaling (e.g., *ANGPT2*, *ANGPT4*, and *ANGPTL3*), which regulate blood vessel development, vascular permeability, inflammation, and angiogenic remodeling (Eklund and Saharinen, 2013). Likewise, miRNAs downregulated in obese-EVs can modulate expression of Vascular Endothelial Growth Factor A (*VEGFA*), and its receptor Kinase Insert Domain Receptor (*KDR*). Activation of VEGF is an important effector of ASC and ASC-derived EV protective effects that induces proliferation and migration of vascular endothelial cells. We have previously shown in experimental renovascular disease that intra-renal delivery of porcine ASC-derived EVs increased expression of VEGF and improved the microvasculature of the post-stenotic kidney (Eirin et al., 2018). However, synthesis of the pro-angiogenic molecule WNT-1 remained unaltered in injured HK2 cells treated with either lean- or obese-EVs, arguing against a major effect of EVs in modulating Wnt signaling in damaged renal tubular cells, despite the RNA sequencing prediction.

MAPK signaling genes can also be targeted by miRNAs both up- and downregulated in obese-EVs compared to lean-EVs. The MAPK signaling pathway is characterized by a family of signaling cascades that elicit cellular responses in response to environmental conditions and stimuli (Chen and Thorner, 2007). p38 can exert protective or deleterious roles in different cell types, which are highly dependent on the type of stimulus. Activation of p38 elicits protective effects during ischemic conditions (Nakano et al., 2000; Fujimoto et al., 2004; Hsu et al., 2007) and plays an important role in the activation of brown adipose tissue (Leiva et al., 2020). However, activation of p38 has been linked to inflammatory cytokine production (Li et al., 2005) and ASC aging (Zhang et al., 2020), and its inhibition potentiates ASC and EV therapy (Zhang et al., 2017; Chen et al., 2020). Our *in vitro* studies revealed that treatment of uninjured HK2 cells with EVs increased expression of p-p38, underscoring the potential of EVs to activate members of MAPK signaling in recipient cells. Contrarily, treatment of injured HK2 cells with either lean- or obese-EVs decreased p-p38 expression, but the effect was more pronounced in lean-EVs compared to obese-EVs, implying that miRNAs packed in obese-EVs might have lower ability to modulate MAPK signaling induced by injurious stimuli.

Our study is limited by a small sample size of relatively young patients. Nevertheless, BMI and waistline circumference were markedly higher in obese vs. lean subjects. Notably, our patients were also morbidly obese. Evidently, despite their young age, obesity sufficed to alter the miRNA cargo of ASC-derived EVs and impair their ability to modulate injury pathways *in vitro*. Our study is also limited by the use of a single EV isolation method (ultracentrifugation) and thus validated sequencing findings following treatment with RNAse. In addition, our results should be interpreted cautiously, given a low mapping rate of the miRNA reads. Further studies are needed to confirm these findings in a larger cohort of patients and in patients with milder obesity, to explore the impact of obesity in the antioxidant effects of EVs, and whether these changes compromise the *in vivo* reparative capacity of EVs in obese subjects. The roles of altered levels of glucose, uric acid, and other abnormal laboratory measures also need to be investigated.

In summary, we found that obesity modifies the size and miRNA cargo of human ASC-derived EVs. Primarily miRNAs enriched in obese-EVs participate in regulation of NF-kB and MAPK signaling, cytoskeleton organization, and apoptosis, whereas those downregulated in obese-EVs are implicated in cell cycle, angiogenesis, and MAPK and Wnt signaling. Importantly, obesity-induced changes in the miRNA cargo of EVs indeed impaired their ability to modulate inflammation, apoptosis, and MAPK signaling *in vitro* in injured renal tubular cells. Therefore, our studies suggest that miRNAs included in EVs may play an important role in regulating cellular injury pathways in ASC-derived EV-treated cells. Therefore, special consideration should be given to subjects with a very high body mass index, because the therapeutic efficacy of EVs derived from ASCs may decrease proportionally.

## Data Availability Statement

The datasets presented in this study can be found in online repositories. The names of the repository/repositories and accession number(s) can be found below: https://figshare.com/, https://figshare.com/articles/dataset/EV-422miRNA/13363376.

## Ethics Statement

The studies involving human participants were reviewed and approved by the First Hospital Affiliated to Jinan University (Guangdong, China) Research Ethics Committee. The patients/participants provided their written informed consent to participate in this study.

## Author Contributions

AE: conception and design, collection and/or assembly of data, data analysis and interpretation, the manuscript writing, and final approval of the manuscript. X-YZ, YL, IS, KJ, and HT: collection and assembly of data, data analysis and interpretation, and the manuscript writing. AL and AW: the manuscript writing and final approval of the manuscript. LL and YM: conception and design, financial support, collection and assembly of data, data analysis and interpretation, the manuscript writing, and final approval of the manuscript. All authors contributed to the article and approved the submitted version.

## Conflict of Interest

LL was an advisor to AstraZeneca and Jonssen Pharmaceutical. The remaining authors declare that the research was conducted in the absence of any commercial or financial relationships that could be construed as a potential conflict of interest.

## Footnotes

1 http://www.targetscan.org/vert_72/
