# Supplementary material for: The Micro-RNA Cargo of Extracellular Vesicles Released by Human Adipose Tissue-Derived Mesenchymal Stem Cells Is Modified by Obesity
Source: Front Cell Dev Biol. 2021 May 20;9:660851. doi: 10.3389/fcell.2021.660851 (PMC8173369; doi:10.3389/fcell.2021.660851)
Supplement: Supplementary file 1 [file Data_Sheet_1.docx]

Supplemental Material

The micro-RNA cargo of extracellular vesicles released by human adipose tissue-derived mesenchymal stem cells is modified by obesity

**Alfonso Eirin^1^, Yu Meng^2^*, Xiang-Yang Zhu^1^, Yongxin Li^1^, Ishran M. Saadiq^1^, Kyra L. Jordan^1^, Hui Tang^1^, Amir Lerman^3^, Andre J. van Wijnen^4^, Lilach O. Lerman^1^***

**Detailed methods:**

*ASC harvesting*

ASCs were isolated from 5-10g of subcutaneous abdominal fat harvested in each individual, and digested in collagenase-H, filtered with 0.2mm syringe filter, and cultured for 3 about weeks in advanced minimal essential medium (GIBCO/Invitrogen, Grand Island, NY, USA) with 5% platelet lysate (PLTmax®, Mill Creek Life Sciences, Rochester, MN) in 37°/5% CO_2_, as previously described (Crespo-Diaz et al., 2011;Eirin et al., 2014;Meng et al., 2017). The seeding density for the stromal vascular fraction was approximately 4x10^6 cells in a T-75 flask, and the subculture seeding density was 2x10^6 cells.

*ASC characterization*

ASCs were characterized using flow cytometry (Amnis FlowSight high-speed cellular imaging, Millipore, Seattle, WA) for the expression of ASC markers (CD73+, CD90+, CD105+, CD34-, CD45-), and trans-differentiation into chondrocytes, adipocytes, and osteocytes (catalog no. SC006; R&D Systems, Minneapolis, MN, https://www.rndsystems.com), as previously described (Eirin et al., 2012;Ebrahimi et al., 2013;Zhu et al., 2013), following criteria set by the International Society for Cellular Therapy (Bourin et al., 2013).

*ASC-derived EV isolation*

The third passage was collected and EVs isolated from supernatants of ASCs by ultracentrifugation, as previously described (Eirin et al., 2014;Eirin et al., 2016;2017b). Buffer preparation included a Wash buffer (M199+20mM HEPES) and 2.42g HEPES to 500mL M199, whereas storage buffer included 2M Sucrose filter sterilized+ 500mM MES pH 6.0 and 6.25 mL sucrose+2 mL MES+water to 50mL. Briefly, the conditioned medium of 10^7^ ASCs was centrifuged to remove debris. Cell-free supernatants were then subjected to a second ultra-centrifugation, washed with M199, and centrifuged one more time, as described below.

1. Grow cells for 80% confluency
2. Remove platelet lysate media and starve using M199 for 48 hours
3. Collect the media into 50mL tubes
4. Spin at 2000g for 25 minutes
5. Pipette the media into the 90ml ultracentrifuge tubes using M199 with 20mM HEPES as a media to balance the tubes
6. Ultracentrifuge at 100000XG for 1 hour: using 1 for a brake and 9 acceleration- this will take approximately 90 minutes. Sorvall ultracentrifuge. T-647.5 Fixed angle rotor.
7. Wash pellet with M199 and ultracentrifuge 100000xg for 60 minutes.
8. Collect and resuspend the pellet in 50uL EV storage buffer
9. Freeze at -80C.

*ASC-derived EV characterization*

Following the minimal standards described by Minimal information for studies of extracellular vesicles 2018 (MISEV2018) guidelines (Thery et al., 2018), EVs were characterized based on the expression of common EV markers by western blotting, electron microscopy (Eirin et al., 2016), and nanoparticle tracking analysis (NTA) (Filipe et al., 2010).

For Western blotting, antibodies against CD9, CD63, and CD81 (Abcam, ab34162, ab193349, and ab33139, respectively) using the following protocol:

BCA sample prep:

1. Using EV isolated previously using ultracentrifugation, dilute the sample 1:10 with ddH2O, but prepare in excess so as to not run out when running duplicates.
2. Take 10uL of the EV sample and mix into a 1.5mL microcentrifuge tube with 90uL ddH2O.
3. From the above dilution, take 25uL and pipette in to wells for BCA assay. Using Thermo Fisher Cat# 23225 Pierce BCA Protein assay kit. Follow kit per manufacturer instructions.

Western blot sample prep:

1. Prepare a set of labeled 1.5mL microcentrifuge tubes.
2. Turn on the hot plate to 100C.
3. According to the results from your BCA protein assay, prepare the samples as indicated.
4. Denature samples for 10 minutes at 100C.

EV western blot:

1. 10 ug of EV protein loaded into gel for western blotting.

2. Samples separated by size using gel electrophoresis- 180V for 45 minutes

3. Proteins transferred to PVDF membrane

4. Membrane blocked overnight at 4C in 5% non-fat dry milk with 0.1% TBST

5. Primary antibodies incubated per manufacturer instructions and washed 3X in TBST.

6. Secondary antibodies incubated per manufacturer instructions and washed 3X in TBST.

7. Incubated with Cat#34096 Supersignal West Femto per manufacturer instructions for 5 minutes at room temperature.

8. Membrane imaged using GE Image Quant LAS4000.

For transmission electron microscopy (TEM), human lean and obese-EVs were stained with 2% uranyl acetate negative staining, and cup-shaped 40-1000 nm structures were identified as EVs in each sample (20 images/sample). Micrographs were taken on a digital electron microscopy (JEOL 1200 EXII, Mayo Clinic electron microscopy core).

EV concentration and size distribution were assessed by NTA analysis (NanoSight NS300). EVs were diluted with PBS to a final volume of 1 ml and subsequently run through a flow-cell top-plate at 25μL/min. Three videos of 120 seconds each were captured for each sample and a minimum of 1,000 tracks were analyzed by the NanoSight Software NTA 2.3.5 (Eirin et al., 2017a). Results from all videos were averaged and concentration and size distribution were compared between human lean- and obese-EVs.

*miRNA sequencing*

The EV miRNA cargo was assessed by miRNA sequencing, as previously described (Li et al., 2020). According to the manufacturer's protocol, total RNA was extracted from EVs using the exoRNeasy Maxi Kit (cat#77044, Qiagen, Germany). Libraries for small RNA sequencing were prepared using the QIAseq miRNA Library Kit (cat#331505, Qiagen, Germany). Briefly, 5μl of total RNA was used as the input for RNA adapter ligation (using 3 and 5 RNA adapters) prior to reverse transcription and PCR amplification with bar-coded primers. The QIAseq library was constructed with a unique molecular index (UMI) which is sequenced during Read 1 and which enables more accurate quantification of miRNA expression levels. The small RNA libraries were concentrated by ethanol precipitation and quantified using a Qubit 2.0 Fluorometer (Thermo Fisher Scientific, USA) prior to sequencing on a HiSeq XTen with read lengths of 75 base pairs and 20 million single-end reads per sample, on average. The library was constructed in the GenCoding Lab (Guangzhou, China), and the sequencing was performed in the Haplox Biotechnology Lab (Shenzhen, China) using an Illumina NGS system (MiSeq Personal Sequencer, NextSequence500, HiSeq 2500). Data were analyzed with CLC (Biomedical) Genomics Workbench. Unaligned FASTQs were used to generate aligned BAMs, raw and normalized known mature miRNA expression counts and predicted novel miRNAs, which were expressed as normalized total reads.

*miRNA quality control*

All samples passed quality control tests before miRNA sequencing. The cDNA concentration was estimated by Qubit measurements with a range from 1.205 to 9.45 ng/µl. Mapped reads content ranged from 5.64% to 9.16% (Tables S1 and S2).

*Table S1: Sample quality inspection*

| Sample | Qubit（ng/μL） | μL | qPCR（nmol/L） | total (10-6 nmol) | Quality inspection |
| --- | --- | --- | --- | --- | --- |
| 1 | 2.83 | 11 | 137.639 | 1514.03 | qualified |
| 2 | 9.45 | 11 | 743.557 | 8179.13 | qualified |
| 3 | 5.45 | 11 | 342.205 | 3764.26 | qualified |
| 4 | 3.9 | 11 | 243.669 | 2680.36 | qualified |
| 5 | 1.345 | 11 | 62.985 | 692.83 | qualified |
| 6 | 1.535 | 11 | 69.112 | 760.24 | qualified |
| 7 | 5.25 | 11 | 282.033 | 3102.37 | qualified |
| 8 | 1.205 | 11 | 54.046 | 594.50 | qualified |
| 9 | 4.435 | 11 | 253.709 | 2790.80 | qualified |
| 10 | 2.105 | 11 | 240.88 | 2649.63 | qualified |

*Table S2: Quality of sequencing data*

| Sample | FastQC_percent_gc | total reads | total reads after QC | Uniquely mapped reads number | Uniquely mapped reads % |
| --- | --- | --- | --- | --- | --- |
| 1 | 61 | 7941695 | 455596 | 40645 | 8.92% |
| 2 | 61 | 5365253 | 1251472 | 96246 | 7.69% |
| 3 | 61 | 7224601 | 1187327 | 73672 | 6.20% |
| 4 | 62 | 4638148 | 502974 | 12389 | 2.46% |
| 5 | 60 | 4806397 | 259167 | 23727 | 9.16% |
| 6 | 61 | 5211931 | 283330 | 22353 | 7.89% |
| 7 | 61 | 7378566 | 939334 | 81654 | 8.69% |
| 8 | 63 | 5353310 | 360696 | 29595 | 8.20% |
| 9 | 63 | 5102071 | 574117 | 35199 | 6.13% |
| 10 | 61 | 4616507 | 525780 | 33214 | 6.32% |

*Bioinformatic analysis*

Differential expression analysis was performed with edgeR2.6.2. miRNAs with fold-change (obese-EVs/lean-EVs)>1.4 (log2=0.5) were considered upregulated, whereas those with fold-change <0.7 (log2=-0.5) were considered downregulated in human obese-EVs versus lean-EVs. Differential p-values were false discovery rate (FDR)-corrected using the Benjamini-Hochberg-Yekutieli procedure (Kim and van de Wiel, 2008).

TargetScan7.2 (http://www.targetscan.org/vert_72/) (Agarwal et al., 2015) and MiRWalk 2.0 (Sticht et al., 2018) were used to identify genes targeted by miRNAs dysregulated in obese-EVs. Target genes were selected via identification of miRNA binding sites within the 5′ untranslated region (UTR), coding sequence (CDS), and 3′ UTR of the human mRNA transcripts. mRNA targets were selected by meeting these criteria: (1) they possessed miRNA binding sites with a binding probability > 0.95; and (2) they were concurrently predicted by miRWalk 3 and the TargetScan algorithm (a filter included in the miRWalk package).

Functional enrichment analysis of miRNA target genes was performed using Gene Set Enrichment Analysis (GSEA, also included in the miRWalk package) (Subramanian et al., 2005), and genes were classified by molecular function (GO: MF), cellular component (GO: CC), and biological process (GO: BP).

*miRNA validation*

To validate the miRNA profile of human EVs, we treated lean- and obese-EVs with 0.2µg/ml of RNAse A (Thermo Fisher Scientific) for 30min at 37°C, and measured expression of randomly selected candidates by quantitative-polymerase chain reaction (qPCR).

*EV functional studies*

To explore the functional implications of obesity-induced changes in the EV cargo, we compared the capacity of human lean- and obese-EVs to modulate inflammation, apoptosis, as well as mitogen-activated protein kinase (MAPK) and Wnt signaling important processes identified in miRNA sequencing in human proximal tubular epithelial cells (HK2 cells). HK2 were co-incubated with 10ng/ml tumor necrosis factor (TNF)-α and 10µM antimycin-A (AMA) for 24hr, a model that mimics renal ischemic injury in vitro (Zhang et al., 2013;Liang et al., 2014), and injured HK2 cells were then co-cultured for 24hr with a pool of 10μg/ml of either lean- or obese-EVs harvested from different subjects (~1 x 10^10 EVs/ml) (Eirin et al., 2020). All experiments were performed in triplicate.

Inflammation was assessed by nuclear translocation of the pro-inflammatory transcription factor nuclear factor (NF)-kB, as previously described (Pawar et al., 2019). Immunofluorescent staining of HK2 cells for NF-kB (abcam, 1:200, Cambridge, MA, USA) and 4′,6′-diamino-2-phenylindole (DAPI, Thermo Fisher Scientific, Waltham, MA) was performed. Nuclear and cytoplasmic localization was assessed and double positive (NFkB+/DAPI+) areas quantified using a computer-aided image analysis program (ZEN® 2012 blue edition; Carl Zeiss SMT, Oberkochen, Germany).

HK2 cell apoptosis was evaluated by terminal deoxynucleotidyl transferase dUTP nick end labeling (TUNEL) staining (abcam, cat#, ab21171, Cambridge, MA) (Eirin et al., 2018). Apoptotic cells were manually counted under fluorescence microscopy, quantified (ZEN®), and identified by co-staining with nuclear (DAPI, blue) and TUNEL (green). Apoptotic cells in were manually counted in 20 images/sample under fluorescence microscopy, and results from all fields were averaged.

Expression of phosphorylated p38 (p-p38) MAPK and WNT-1 was assessed by Western blotting (Cell signaling, cat#: 9212, 1:1000 and abcam cat#: ab15251, 1:100, respectively).

*Statistical analysis*

Statistical analysis was performed using JMP software package version 14 (SAS Institute, Inc., Cary, NC, USA). The Shapiro–Wilk test was used to test for deviation from normality. Normally distributed data, expressed as mean±SD, were compared using Student t-test. Non-normally distributed data were expressed as medium (range) and compared using nonparametric (Kruskal Wallis) tests. A P value ≤0.05 was considered statistically significant.

**References**

Agarwal, V., Bell, G.W., Nam, J.W., and Bartel, D.P. (2015). Predicting effective microRNA target sites in mammalian mRNAs. *Elife* 4.

Bourin, P., Bunnell, B.A., Casteilla, L., Dominici, M., Katz, A.J., March, K.L., Redl, H., Rubin, J.P., Yoshimura, K., and Gimble, J.M. (2013). Stromal cells from the adipose tissue-derived stromal vascular fraction and culture expanded adipose tissue-derived stromal/stem cells: a joint statement of the International Federation for Adipose Therapeutics and Science (IFATS) and the International Society for Cellular Therapy (ISCT). *Cytotherapy* 15**,** 641-648.

Crespo-Diaz, R., Behfar, A., Butler, G.W., Padley, D.J., Sarr, M.G., Bartunek, J., Dietz, A.B., and Terzic, A. (2011). Platelet lysate consisting of a natural repair proteome supports human mesenchymal stem cell proliferation and chromosomal stability. *Cell Transplant* 20**,** 797-811.

Ebrahimi, B., Eirin, A., Li, Z., Zhu, X.Y., Zhang, X., Lerman, A., Textor, S.C., and Lerman, L.O. (2013). Mesenchymal stem cells improve medullary inflammation and fibrosis after revascularization of swine atherosclerotic renal artery stenosis. *PLoS One* 8**,** e67474.

Eirin, A., Ferguson, C.M., Zhu, X.Y., Saadiq, I.M., Tang, H., Lerman, A., and Lerman, L.O. (2020). Extracellular vesicles released by adipose tissue-derived mesenchymal stromal/stem cells from obese pigs fail to repair the injured kidney. *Stem Cell Res* 47**,** 101877.

Eirin, A., Riester, S.M., Zhu, X.Y., Tang, H., Evans, J.M., O'brien, D., Van Wijnen, A.J., and Lerman, L.O. (2014). MicroRNA and mRNA cargo of extracellular vesicles from porcine adipose tissue-derived mesenchymal stem cells. *Gene* 551**,** 55-64.

Eirin, A., Zhu, X.Y., Jonnada, S., Lerman, A., Van Wijnen, A.J., and Lerman, L.O. (2018). Mesenchymal Stem Cell-Derived Extracellular Vesicles Improve the Renal Microvasculature in Metabolic Renovascular Disease in Swine. *Cell Transplant* 27**,** 1080-1095.

Eirin, A., Zhu, X.Y., Krier, J.D., Tang, H., Jordan, K.L., Grande, J.P., Lerman, A., Textor, S.C., and Lerman, L.O. (2012). Adipose tissue-derived mesenchymal stem cells improve revascularization outcomes to restore renal function in swine atherosclerotic renal artery stenosis. *Stem Cells* 30**,** 1030-1041.

Eirin, A., Zhu, X.Y., Puranik, A.S., Tang, H., Mcgurren, K.A., Van Wijnen, A.J., Lerman, A., and Lerman, L.O. (2017a). Mesenchymal stem cell-derived extracellular vesicles attenuate kidney inflammation. *Kidney Int* 92**,** 114-124.

Eirin, A., Zhu, X.Y., Puranik, A.S., Woollard, J.R., Tang, H., Dasari, S., Lerman, A., Van Wijnen, A.J., and Lerman, L.O. (2016). Comparative proteomic analysis of extracellular vesicles isolated from porcine adipose tissue-derived mesenchymal stem/stromal cells. *Sci Rep* 6**,** 36120.

Eirin, A., Zhu, X.Y., Puranik, A.S., Woollard, J.R., Tang, H., Dasari, S., Lerman, A., Van Wijnen, A.J., and Lerman, L.O. (2017b). Integrated transcriptomic and proteomic analysis of the molecular cargo of extracellular vesicles derived from porcine adipose tissue-derived mesenchymal stem cells. *PLoS One* 12**,** e0174303.

Filipe, V., Hawe, A., and Jiskoot, W. (2010). Critical evaluation of Nanoparticle Tracking Analysis (NTA) by NanoSight for the measurement of nanoparticles and protein aggregates. *Pharm Res* 27**,** 796-810.

Kim, K.I., and Van De Wiel, M.A. (2008). Effects of dependence in high-dimensional multiple testing problems. *BMC Bioinformatics* 9**,** 114.

Liang, X., Chen, Y., Zhang, L., Jiang, F., Wang, W., Ye, Z., Liu, S., Yu, C., and Shi, W. (2014). Necroptosis, a novel form of caspase-independent cell death, contributes to renal epithelial cell damage in an ATP-depleted renal ischemia model. *Mol Med Rep* 10**,** 719-724.

Meng, Y., Eirin, A., Zhu, X.Y., Tang, H., Chanana, P., Lerman, A., Van Wijnen, A.J., and Lerman, L.O. (2017). Obesity-induced mitochondrial dysfunction in porcine adipose tissue-derived mesenchymal stem cells. *J Cell Physiol*.

Pawar, A.S., Eirin, A., Krier, J.D., Woollard, J.R., Zhu, X.Y., Lerman, A., Van Wijnen, A.J., and Lerman, L.O. (2019). Alterations in genetic and protein content of swine adipose tissue-derived mesenchymal stem cells in the metabolic syndrome. *Stem Cell Res* 37**,** 101423.

Thery, C., Witwer, K.W., Aikawa, E., Alcaraz, M.J., Anderson, J.D., Andriantsitohaina, R., Antoniou, A., Arab, T., Archer, F., Atkin-Smith, G.K., Ayre, D.C., Bach, J.M., Bachurski, D., Baharvand, H., Balaj, L., Baldacchino, S., Bauer, N.N., Baxter, A.A., Bebawy, M., Beckham, C., Bedina Zavec, A., Benmoussa, A., Berardi, A.C., Bergese, P., Bielska, E., Blenkiron, C., Bobis-Wozowicz, S., Boilard, E., Boireau, W., Bongiovanni, A., Borras, F.E., Bosch, S., Boulanger, C.M., Breakefield, X., Breglio, A.M., Brennan, M.A., Brigstock, D.R., Brisson, A., Broekman, M.L., Bromberg, J.F., Bryl-Gorecka, P., Buch, S., Buck, A.H., Burger, D., Busatto, S., Buschmann, D., Bussolati, B., Buzas, E.I., Byrd, J.B., Camussi, G., Carter, D.R., Caruso, S., Chamley, L.W., Chang, Y.T., Chen, C., Chen, S., Cheng, L., Chin, A.R., Clayton, A., Clerici, S.P., Cocks, A., Cocucci, E., Coffey, R.J., Cordeiro-Da-Silva, A., Couch, Y., Coumans, F.A., Coyle, B., Crescitelli, R., Criado, M.F., D'souza-Schorey, C., Das, S., Datta Chaudhuri, A., De Candia, P., De Santana, E.F., De Wever, O., Del Portillo, H.A., Demaret, T., Deville, S., Devitt, A., Dhondt, B., Di Vizio, D., Dieterich, L.C., Dolo, V., Dominguez Rubio, A.P., Dominici, M., Dourado, M.R., Driedonks, T.A., Duarte, F.V., Duncan, H.M., Eichenberger, R.M., Ekstrom, K., El Andaloussi, S., Elie-Caille, C., Erdbrugger, U., Falcon-Perez, J.M., Fatima, F., Fish, J.E., Flores-Bellver, M., Forsonits, A., Frelet-Barrand, A., et al. (2018). Minimal information for studies of extracellular vesicles 2018 (MISEV2018): a position statement of the International Society for Extracellular Vesicles and update of the MISEV2014 guidelines. *J Extracell Vesicles* 7**,** 1535750.

Zhang, L., Jiang, F., Chen, Y., Luo, J., Liu, S., Zhang, B., Ye, Z., Wang, W., Liang, X., and Shi, W. (2013). Necrostatin-1 attenuates ischemia injury induced cell death in rat tubular cell line NRK-52E through decreased Drp1 expression. *Int J Mol Sci* 14**,** 24742-24754.

Zhu, X.Y., Urbieta-Caceres, V., Krier, J.D., Textor, S.C., Lerman, A., and Lerman, L.O. (2013). Mesenchymal stem cells and endothelial progenitor cells decrease renal injury in experimental swine renal artery stenosis through different mechanisms. *Stem Cells* 31**,** 117-125.
